# Supplementary material for: Describing the Sensory Complexity of Italian Wines: Application of the Rate-All-That-Apply (RATA) Method
Source: Foods. 2022 Aug 11;11(16):2417. doi: 10.3390/foods11162417 (PMC9407563; doi:10.3390/foods11162417)
Supplement: Supplementary file 1 [file foods-11-02417-s001.zip › Supplementary Figure S1.pdf]

Supplementary Figure S1. First and second dimensions of MFA depicting wine samples configuration from the two separate replicates.

The first two dimensions explained 44.39 % of total explained variance. The two replicates of all wine samples evaluated were located near in the bidimensional space. Moreover, dimension 1 (explained variance 23.79 %) separated all red wines from the rest of the samples, while dimension 2 (explained variance 20.60 %) distinguished white wines from sweet wines (Moscato d'Asti and Sangue di Giuda wines in the lower part of the space), indicating a good panel discriminating ability.

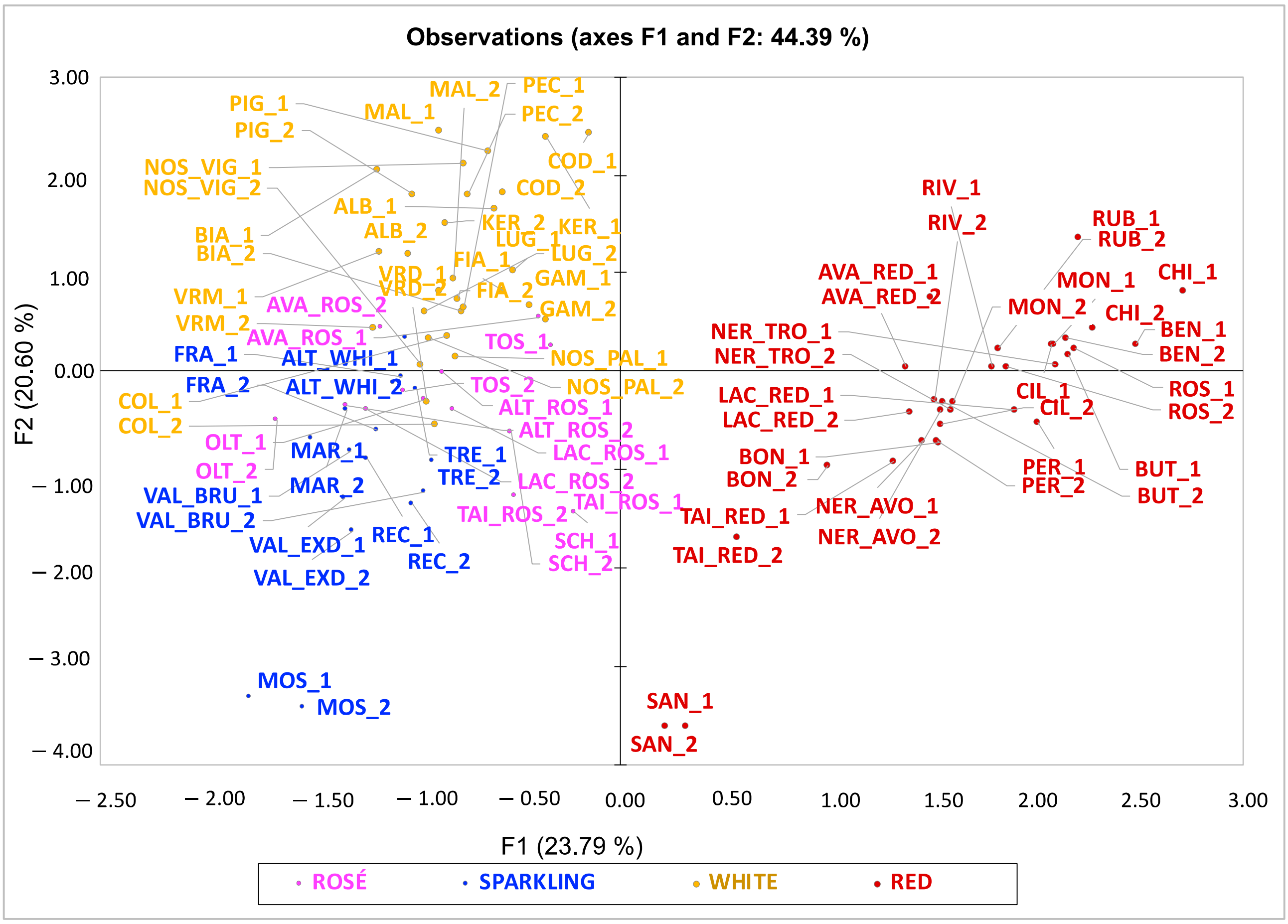

WHITE WINES

ALB: Romagna Albana DOCG; BIA: Ischia DOC Biancolella; COD: Coda di Volpe DOC; COL: Collio Ribolla Gialla DOC; FIA: Fiano di Avellino DOCG; GAM: Gambellara Classico DOC; KER: Vigneti delle Dolomiti IGT, Kerner; LUG: Lugana Riserva DOC; MAL: Collio Malvasia DOC; NOS_PAL: Nosiola Palustella Trentino DOC; NOS_VIG: Vigneti delle Dolomiti IGT, Nosiola; PEC: Pecorino DOP; PIG: Colli Bolognesi Pignoletto Superiore DOCG; VRD: Verdicchio Dei Castelli Di Jesi DOC Classico Superiore; VRM: Vermentino DOC.

RED WINES

AVA_RED: Valsusa DOC; BEN: Benaco Bresciano IGT; BON: Bonarda dell'Oltrepò Pavese DOC; BUT: Buttafuoco dell'Oltrepò Pavese DOC; CHI: Chianti Superiore DOCG; CIL: Maremma Toscana DOC; LAC_RED: Lacrima di Morro d'Alba DOC; MON: Colline Teremane Montepulciano d'Abruzzo DOCG; NER_AVO: Nero d'Avola Menfi DOC; NER_TRO: Cacc'e Mmitte di Lucera DOC; PER: Perricone Terre siciliane IGT; RIV: Riviera del Garda Classico DOC; ROS: Rossese Di Dolceacqua Superiore DOC; RUB: Rubicone Centesimino IGT; SAN: Sangue di Giuda dell'Oltrepò Pavese DOC; TAI_RED: Colli Berici Tai Rosso DOC.

SPARKLING WINES

ALT_WHI: Alta Langa DOCG Extra Brut; FRA: Franciacorta DOCG Brut; MAR: Marche IGT; MOS: Moscato d'Asti DOCG; REC: Recioto Spumante Metodo Classico DOCG; TRE: Trento Metodo Classico DOCG millesimato; VAL_BRU: Valdobbiadene DOCG Brut; VAL_EXD: Valdobbiadene DOCG Extra Dry.

ROSE' WINES

ALT_ROS: Alta Langa DOCG; AVA_ROS: Vino Rosato Frizzante; LAC_ROS: Spumante Rosato Brut; OLT: Oltrepò Pavese Metodo Classico Pinot Nero Rosé DOCG; SCH: Vigneti delle Dolomiti IGT, Schiava; TAI_ROS: Colli Berici Tai Rosato DOC; TOS: Toscana IGT.
