# Supplementary material for: Describing the Sensory Complexity of Italian Wines: Application of the Rate-All-That-Apply (RATA) Method
Source: Foods. 2022 Aug 11;11(16):2417. doi: 10.3390/foods11162417 (PMC9407563; doi:10.3390/foods11162417)
Supplement: Supplementary file 1 [file foods-11-02417-s001.zip › supplementary tables S 1-3.pdf]

Supplementary Table S1. Complete list of descriptors used for the sensory characterisation of the wine samples (W=white, R=red, S=sparkling, Rs=rosé) and reference standards (pure aromas/composition in wine) used for odour/flavour, taste and mouthfeel identification tasks in the training phase.

**SENSORY DIMENSION**	**MACROCATEGORY**	**STIMULUS**	**Wine**	**Pure aroma**	**Reference standard in table wine[a]**	**Bibliography**
**Odour/flavour**	CITRUS	Lemon	W, S, Rs	Le Nez du Vin®	5 mL fresh lemon juice and peel in 25 mL table wine	[35]
		Orange	W, S, Rs	Le Nez du Vin®	5 mL fresh orange juice and peel in 25 mL table wine	
		Grapefruit	W, S, Rs	Le Nez du Vin®	5 mL fresh grapefruit juice and peel in 25 mL table wine	[30]
	TROPICAL FRUITS	Pineapple	W, S, Rs	Le Nez du Vin®	2-4 mL pineapple juice in 25 mL table wine	[35]
		Banana	W, S, Rs	Le Nez du Vin®	1 slice (1 cm thick) fresh banana in 25 mL table wine	[35]
		Litchi	W, S, Rs	Le Nez du Vin®		
		Melon	W, S, Rs	Le Nez du Vin®	1-piece fresh ripe cantaloupe (20 mm cube) in 25 mL table wine	[30]
		Passion fruit	S, Rs		1-2 blended passion fruit seeds in 25 mL table wine	
	RED FRUITS	Strawberry	R, Rs	Le Nez du Vin®	1-2 crushed fresh or frozen strawberries in 25 mL table wine	[30]
		Cherry	R, Rs	Le Nez du Vin®	10 mL brine of canned cherries in 25 mL table wine	[30]
		Pomegranate	R, Rs		1-2 pulsed pomegranate arils in 25 mL table wine	
		Sour black cherry	R		10 mL sour cherry syrup in 25 mL table wine	[34]
	WILD BERRIES	Currant	R, Rs	Le Nez du Vin®	1-2 crushed fresh or frozen currant berries in 25 mL table wine	[35]
		Raspberry	R, Rs	Le Nez du Vin®	1-2 crushed fresh or frozen raspberries in 25 mL table wine	[30]
		Blackberry	R, Rs	Le Nez du Vin®	1-2 crushed fresh or frozen blackberries in 25 mL table wine	[35]
		Blueberry	R	Le Nez du Vin®	1-2 crushed fresh or frozen blueberries in 25 mL table wine	

		Mulberry	R	Le Nez du Vin®		
	FRUIT TREE	Apple	W, S, Rs	Le Nez du Vin®	1 slice fresh apple or 5 mL of apple juice in 25 mL table wine	[35]
		Pear	W, S, Rs	Le Nez du Vin®	1 slice of fresh pear or 5 mL of pear juice in 25 mL table wine	
		Quince	W, S, Rs	Le Nez du Vin®		
		Moscato grape	W, S, Rs	Le Nez du Vin®		
		Peach	W, S, Rs	Le Nez du Vin®	15-20 mL peach juice in 25 mL table wine	[35]
		Apricot	W, S, Rs	Le Nez du Vin®	15-20 mL apricot nectar in 25 mL table wine	[30]
	NUTS	Almond	W, S, R, Rs	Le Nez du Vin®	1 drop of almond flavour extract (Rebecchi F.lli Valtrebbia SpA, Piacenza, IT) in 100 mL table wine	[35]
		Walnuts	W, S, R, Rs	Le Nez du Vin®	1-2 walnuts, crushed (no wine)	[30]
		Hazelnut	W, S, R, Rs	Le Nez du Vin®	1-2 hazelnuts, ground (no wine)	[35]
	DRIED/BAKED FRUIT	Dried prune	S, R, Rs	Le Nez du Vin®	1-2 mL prune juice in 25 mL table wine	[35]
		Raisins	W, S, Rs		5-8 pressed raisin grapes in 25 mL table wine	[35]
		Dried figs	W, S, Rs		1-2 fig or 5-10 mL canned fig juice in 25 mL table wine	[35]
		Fruit jam	R, Rs		1 teaspoon of strawberry jam in 25 mL table wine	[35]
		Ripe fruit	S, Rs		40 g apple puree in 300 mL of table wine	[31]
	WHITE FLORAL	Hawthorn	W, S, Rs	Le Nez du Vin®	1-2 dried hawthorn flowers in 25 mL white table wine	
		Acacia	W, S, Rs	Le Nez du Vin®		
		Chamomile	W, S, Rs	Tasterplace©		
		Linden	W, S, Rs	Le Nez du Vin®	1-2 dried linden flowers in 25 mL table wine	
		Jasmine	W, S, Rs		2 drops of jasmine essential oil (Olio essenziale Gelsomino 20%, Laboratorio Nonna Ortica, Verbania, Italy) in 100 mL table wine	[33]

		Orange blossoms	R, S, Rs		1 crushed orange blossom in 25 ml table wine	[30]
	RED FLORAL	Rose	W, R, Rs	Le Nez du Vin®	1-2 rose petals in 25 mL table wine	[35]
		Violet	R, Rs	Le Nez du Vin®	1-2 violet petals in 25 mL table wine	[30]
		Dried flowers	R, S, Rs		1-2 crushed dried flowers in 25 mL table wine	
	VEGETATIVE	Sage	W, S, R, Rs	Tasterplace©	1 crushed sage leaf in 25 mL table wine	
		Hay	W, S, R, Rs	Le Nez du Vin®	Several pieces of hay, finely cut (no wine)	[30]
		Fresh cut grass	S, R, Rs	Tasterplace©	1 shredded 20-mm blade of green grass in 25 mL table wine	[30]
		Marjoram	S, Rs	Tasterplace©	1 crushed marjoram leaf in 25 mL table wine	
		Rosemary	W, S, R, Rs	Tasterplace©	1 crushed rosemary leaf in 25 mL table wine	
		Oregano	R	Tasterplace©		
		Bell pepper	R	Le Nez du Vin®	slice of bell pepper soaked 30 min in 25 mL table wine	[30]
	BALSAMIC	Thyme	W, S, R, Rs	Le Nez du Vin®		
		Eucalyptus	W, S, R, Rs		1 crushed eucalyptus leaf in 25 mL table wine	[30]
		Mint	W, S, R, Rs		1 crushed mint leaf or 1 drop mint extract in 25 mL table wine	[30]
		Anise	S, R, Rs	Tasterplace©	1 drop anise extract in 50 mL table wine	[30]
	SPICES	Vanilla	W, S, R, Rs	Le Nez du Vin®	1-2 drops of vanilla flavour extract (Rebecchi F.lli Valtrebbia SpA, Piacenza, IT) in 25 mL table wine	[35]
		Saffron	W, S, Rs	Le Nez du Vin®		
		Pepper	R	Le Nez du Vin®	2-3 grains ground black pepper in 25 mL table wine	[35]
		Liquorice	R	Le Nez du Vin®	1 small piece of licorice stick in 25 mL table wine	[35]

		Cloves	R	Le Nez du Vin®	1 clove left to infuse for about 2 hours in 25 mL table wine	[35]
		Cinnamon	R	Le Nez du Vin®	½ teaspoon of cinnamon powder in 250 mL table wine	[32]
		Juniper	R		1-2 juniper berries in 25 mL table wine	
	EARTHY	Mushroom	W, S, R, Rs	Le Nez du Vin®	1 small mushroom, finely sliced in 10 mL table wine	[30]
		Musk	W, S, R, Rs	Le Nez du Vin®		
		Truffle	W, S, R, Rs	Le Nez du Vin®		
		Lees	W, S, R, Rs	Le Nez du Vin®		
	ROASTED	Toasted bread	W, S, R, Rs	Le Nez du Vin®		
		Smoke	R	Le Nez du Vin®	1 drop smoky flavour extract (Flavourart, Oleggio, IT) in 150 mL table wine	[30]
		Chocolate	R	Le Nez du Vin®	1/2 teaspoon powdered cocoa in 25 mL table wine	[30]
		Coffee	R	Le Nez du Vin®	2-3 ground coffee beans in 25 mL table wine	[35]
		Tobacco	R		3-4 flakes of tobacco (least aromatic possible) in 25 mL table wine	[30]
	WOODY	Oak	S, R, Rs	Tasterplac e©	2-3 drops oak flavour extract (Flavourart, Oleggio, IT) in 25 mL table wine	[30]
		Cedar wood	W, S	Le Nez du Vin®	1 drop cedar essential oil (Olio essenziale legno di cedro, Laboratorio Nonna Ortica, Verbania, Italy) in 25 mL table wine	[30]
		Pine	W, S	Le Nez du Vin®	2-3 drops pine essential oil (Olio essenziale Pino Silvestre, Laboratorio Nonna Ortica, Verbania, Italy) in 25 mL table wine	[30]
	CARAMELISE D	Caramel	W, S, R, Rs	Le Nez du Vin®	2 mL of caramel aroma (Funcakes Caramel Pasta aromatizzante, Newcakes, NL) in 300 mL table wine	[31]
		Butter	W, S, R, Rs	Le Nez du Vin®	1 drop butter flavour extract (Flavourart, Oleggio, IT) in 100 mL table wine	[30]
		Honey	W, S, Rs	Le Nez du Vin®	5-8 mL chestnut honey in 25 mL table wine	[31]
		Candied citron	W	Le Nez du Vin®		
	YEAST	Yeast	S, Rs		1/4 teaspoon of active-dried baker’s yeast in 100 mL table wine	[74]

		Bread crust	S, Rs	Tasterplace©		
	ETHEREAL	Dust	S, Rs	Tasterplace©		
		Flint stone	W, S, Rs		spark wheel of a cigarette lighter, spun (no wine)	
		Solvent	W, S, Rs		2 mL of acetone in 300 mL table wine	[31]
		Fuel	W, S, Rs		1 drop kerosene in 150 mL table wine	[30]
	ANIMAL	Leather	R	Le Nez du Vin®		
**Taste**		Sweet	S, R, Rs		78 g of fructose in 300 mL table wine	[31]
		Sour	W, S, R, Rs		0.20 g of tartaric acid in 300 mL table wine	[31]
		Bitter	W, S, R, Rs		0.8 g of caffein in 500 mL table wine	[34]
		Salty	W, S, Rs		50 g of sodium chloride in 500 mL table wine	
**Tactile sensations**		Astringency	S, R, Rs		1.5 g of tannin in 750 mL table wine	[34]
		Alcohol	W, S, R, Rs		45 mL of 95% ethyl alcohol in 300 mL table wine	[31]
		Body	W, S, R, Rs		3 mL of glycerol in 500 mL table wine	[34]

[a] white table wine:Tavernello Classico, vino bianco d'Italia (Cooperativa Agricola Caviro, Feanza, Italy); red table wine: Tavernello Classico, vino rosso d'Italia (Cooperativa Agricola Caviro, Feanza, Italy).

All reference standards in wine were infused at least 12h at room temperature.

Supplementary Table S2. Aqueous solutions used for the training phase.

Tasks	Stimulus	Reference standards	Concentration (g/L)
Taste and tactile sensations identification [37]	Sour	Citric acid monohydrate	1.20
	Bitter	Caffeine monohydrate	0.54
	Salty	Anhydrous sodium chloride	4.00
	Sweet	Sucrose	24.00
	Astringent	Catechin	2.00
Ranking [39]	Sour	Tartaric acid	(0.25 g/L; 0.5 g/L; 0.75 g/L; 1 g/L)
	Bitter	Quinine sulphate	(0.5 mg/L; 2.5 mg/L; 4.5 mg/L; 6.5 mg/L)
	Sweet	Sucrose	(2 g/L; 6 g/L; 10 g/L; 14 g/L)
	Astringent	Tannic acid	(0.5 g/L; 0.75 g/L; 1 g/L; 1.25 g/L)
	Body	Glycerol	(0 g/L; 3 g/L; 6 g/L; 9 g/L)

Reference chemical details: Citric acid monohydrate, Caffeine monohydrate, Anhydrous sodium chloride, Sucrose, Catechin, Tartaric acid, Quinine sulphate and Tannic acid (Sigma Aldrich srl, Milano, Italy); Glycerol (Glicerolo vegetale F.U., Marco Viti Farmaceutici spa, Como, Italy).

Supplementary Table S3. Mean RI values by judge for white, red and sparkling white and rosé wines. For each judge and wine category, the minimum value registered (Min.) as well as the standard deviation (SD) are reported between brackets.

Judge	White wines	Red wines	Sparkling white & rosé wines
**C002**	**0.843**<br>(Min. 0.778; SD 0.032)	**0.866**<br>(Min. 0.783; SD 0.036)	**0.877**<br>(Min. 0.817; SD 0.030)
**C003**	**0.768**<br>(Min. 0.704; SD 0.035)	**0.724**<br>(Min. 0.587; SD 0.076)	**0.706**<br>(Min. 0.608; SD 0.062)
**C004**	**0.706**<br>(Min. 0.580; SD 0.077)	**0.749**<br>(Min. 0.609; SD 0.068)	**0.744**<br>(Min. 0.558; SD 0.066)
**C005**	**0.758**<br>(Min. 0.543; SD 0.077)	**0.822**<br>(Min. 0.717; SD 0.056)	**0.849**<br>(Min. 0.792; SD 0.042)
**C006**	**0.702**<br>(Min. 0.605; SD 0.055)	**0.715**<br>(Min. 0.620; SD 0.068)	**0.757**<br>(Min. 0.683; SD 0.064)
**C007**	**0.814**<br>(Min. 0.716; SD 0.061)	**0.848**<br>(Min. 0.739; SD 0.066)	**0.866**<br>(Min. 0.808; SD 0.037)
**C008**	**0.735**<br>(Min. 0.605; SD 0.081)	**0.709**<br>(Min. 0.554; SD 0.064)	**0.743**<br>(Min. 0.617; SD 0.084)
**C009**	**0.709**<br>(Min. 0.531; SD 0.107)	**0.715**<br>(Min. 0.630; SD 0.052)	**0.681**<br>(Min. 0.550; SD 0.057)
**C010**	**0.789**<br>(Min. 0.667; SD 0.064)	**0.840**<br>(Min. 0.750; SD 0.052)	**0.843**<br>(Min. 0.750; SD 0.045)
**C011**	**0.817**<br>(Min. 0.716; SD 0.046)	**0.833**<br>(Min. 0.761; SD 0.043)	**0.852**<br>(Min. 0.808; SD 0.035)
**C012**	**0.865**<br>(Min. 0.765; SD 0.037)	**0.868**<br>(Min. 0.783; SD 0.046)	**0.861**<br>(Min. 0.800; SD 0.030)
**C013**	**0.775**<br>(Min. 0.654; SD 0.069)	**0.815**<br>(Min. 0.739; SD 0.043)	**0.853**<br>(Min. 0.775; SD 0.049)
**Mean RI**	**0.773**<br>(Min. 0.702; SD 0.054)	**0.792**<br>(Min. 0.709; SD 0.064)	**0.803**<br>(Min. 0.681; SD 0.071)
